# Lectins Offer New Perspectives in the Development of Macrophage-Targeted Therapies for COPD/Emphysema

**DOI:** 10.1371/journal.pone.0056147

**Published:** 2013-02-18

**Authors:** Violet R. Mukaro, Johan Bylund, Greg Hodge, Mark Holmes, Hubertus Jersmann, Paul N. Reynolds, Sandra Hodge

**Affiliations:** 1 Department of Thoracic Medicine, Royal Adelaide Hospital and Lung Research Laboratory, Hanson Institute, Adelaide, South Australia, Australia; 2 Department of Rheumatology and Inflammation Research, The Sahlgrenska Academy at University of Gothenburg, Gothenburg, Sweden; 3 Department of Medicine, University of Adelaide, Adelaide, South Australia, Australia; National Institute of Infectious Diseases, Japan

## Abstract

We have previously shown that the defective ability of alveolar macrophages (AM) to phagocytose apoptotic cells (‘efferocytosis’) in chronic obstructive pulmonary disease/emphysema (COPD) could be therapeutically improved using the C-type lectin, mannose binding lectin (MBL), although the exact mechanisms underlying this effect are unknown. An S-type lectin**,** galectin-3, is also known to regulate macrophage phenotype and function, via interaction with its receptor CD98. We hypothesized that defective expression of galectin/CD98 would be associated with defective efferocytosis in COPD and that mechanisms would include effects on cytoskeletal remodeling and macrophage phenotype and glutathione (GSH) availability. Galectin-3 was measured by ELISA in BAL from controls, smokers and current/ex-smokers with COPD. CD98 was measured on AM using flow cytometry. We assessed the effects of galectin-3 on efferocytosis, CD98, GSH, actin polymerisation, rac activation, and the involvement of PI3K (using β-actin probing and wortmannin inhibition) *in vitro* using human AM and/or MH-S macrophage cell line. Significant decreases in BAL galectin-3 and AM CD98 were observed in BAL from both current- and ex-smoker COPD subjects vs controls. Galectin 3 increased efferocytosis via an increase in active GTP bound Rac1. This was confirmed with β-actin probing and the role of PI3K was confirmed using wortmannin inhibition. The increased efferocytosis was associated with increases in available glutathione and expression of CD98. We provide evidence for a role of airway lectins in the failed efferocytosis in COPD, supporting their further investigation as potential macrophage-targeted therapies.

## Background

Chronic Obstructive Pulmonary Disease/emphysema (COPD) is a disease that is poorly managed with currently available therapies. The World Health Organization estimates that 80 million people have moderate to severe COPD and this will be the third leading cause of death world-wide by 2030 [1). There still exist gaps in the understanding of the pathogenic mechanisms of the disease; however, we have shown a significant defect in the ability of pulmonary macrophages to phagocytose apoptotic airway epithelial cells (defective ***efferocytosis***) [2]–[4], which contributes to an excess of apoptotic material present in the airways of these patients [5]. This uncleared material can then undergo secondary necrosis and perpetuate chronic inflammation [5], [6]. We have also demonstrated impaired phagocytosis of bacteria in COPD; an important finding given the increased bacterial colonization and increased susceptibility for infection/exacerbation [7].

Lectins are soluble carbohydrate-binding proteins that include C-type (lung surfactants and mannose binding lectin (MBL)), S-type (galectins), L-type, heparin binding proteins and pentraxins. They contain carbohydrate recognition domains (CRD) and are traditionally recognised for their roles in recognition of pathogen-associated molecular patterns (PAMPs) and facilitation of pathogen clearance [8]–[11]. More recently we and others have shown that lectins also have the ability to facilitate phagocytosis of apoptotic cells [12], [13]. MBL, for example, has been shown to recognize nucleic acids including fragmented DNA on apoptotic cells, products of tissue damage (eg, heat shock proteins, cell membrane material) [8]. We have shown that defective efferocytosis in COPD (and other chronic inflammatory lung diseases, including bronchiolitis obliterans syndrome following lung transplantation) is associated with a decrease in airway levels of several C-type lectins including MBL and surfactants [14], [15], although levels of S-type lectins have not been assessed in COPD. We further showed that MBL administration to smoking mice improved efferocytosis and reduced inflammation; however, the precise mechanisms for this effect are unclear [16].

Both MBL and galectin-3 have been shown to have effects on components of the actin reorganization pathway that occurs during the phagocytic process. We showed that the pro-efferocytosis effects of MBL were accompanied by an increase in intracellular Rac1/2/3 [16]. Galectin-3 has also been shown to activate phosphatidylinositol 3-kinase (PI3K), which has a well-recognized role in the reorganization of the actin skeleton and activation of Rac, during myelin phagocytosis in microglia [17].

Lectins may also contribute to intracellular production of glutathione (GSH), an antioxidant present in the lung that is important for effective efferocytosis [18], [19]. The availability of extracellular GSH is essential to maintain intracellular GSH homeostasis; when GSH availability is limited, cellular functions including efferocytosis and respiratory burst are reduced [18]. GSH is produced following transport of its precursor amino acid, cystine, into the cell via its transporter- xCT/CD98 [20]. Both MBL and galectin-3 bind to CD98 [8], [21], and the interaction of galectin-3 and CD98 has been shown to induce an ‘M2’ alternatively activated macrophage phenotype (with an improved efferocytosis ability) [18], [21]. It is therefore possible that low levels of galectin-3, or reduced expression of CD98, could compromise the levels of GSH and subsequent efferocytosis ability of airway macrophages, and play a role in the defective efferocytosis observed in COPD.

We firstly measured galectin-3 and macrophage CD98 expression in the airways of well-categorized controls, smokers and COPD groups. We then determined whether administration of exogenous galectin-3 would improve efferocytosis *in vitro*, and investigated potential mechanisms for this effect including increased GSH availability, effects on macrophage phenotype, actin polymerisation, Rac1 activation, and the involvement of PI3K.

## Methods

### Bronchoscopy and Preparation of Alveolar Macrophages (AM)

Ethical approval was granted by the Royal Adelaide Hospital Ethics Committee and written informed consent was obtained for each patient or control recruited for the study. Flexible bronchoscopy was performed and bronchoalveolar lavage samples (BAL) obtained according to recommendations by the American Thoracic Society as previously reported [2]–[4]. The diagnosis of COPD was established using the GOLD criteria (FEV1/FVC <70%) with clinical correlation [22].

Alveolar macrophages from BAL were prepared as previously reported [2]–[4]. Briefly, macrophage cell counts in BAL were adjusted to 4×10^5^/mL in RPMI 1640 media, supplemented with 10% foetal calf serum, 1% weight per volume L-Glutamine and penicillin/streptomycin (Gibco BRL, Berlin, Germany) (culture medium). One mL aliquots were then adhered to 24 well culture plates for 2 h, then fluid removed and the purified macrophages gently recovered using ice-cold culture medium and gentle pipetting. We have previously shown that this process results in no significant changes in apoptosis or macrophage function (data not shown). Total and differential cell counts were obtained and the macrophage cell counts adjusted to 4×10^5^/ml in culture medium Macrophages were purified by adhesion to plastic as previously reported [2]–[4].

### Human Subjects

BAL galectin-3 was evaluated for (i) never-smoker controls (n = 15) (ii) healthy smoker controls (n = 12) (iii) healthy ex-smoker controls (n = 8) (iv) subjects with moderate severity COPD (30; 18 current smokers and 12 ex-smokers) (Table 1). All ex-smokers had ceased smoking for at least 12 months.

**Table 1:** BAL patient demographics.

	N [M/F]	Age(Years)	Pack-Years	FEV1(% Pred)	FEV1/FVC	Volume (mL)	Volume(% instilled)	WCC(x10^9^/L)	Mac(%)	Lymph(%)	Neut(%)
**Control** **never-smokers**	[7/8]	49(27–72)	0	99(81–134)	83(74–98)	72(42–88)	48(28–59)	0.2(0.04–0.75)	82(27–93)	10 (4–26)	7 (0–25)
**Control** **ex-smokers**	[5/3]	59(33–72)	20(8–30)*	103(65–126)	82(72–103)	70(43–81)	47(29–54)	0.13(0.06–0.31)	85(70–95)	9 (2–28)	6 (1–14)
**Control** **smokers**	[7/5]	56(26–66)	40(7–120)*	83(74–97)	75(70–88)	63(26–85)	40(17–57)	1.18(0.14–7.6)	83(61–98)	12 (0–33)	7 (1–33)
**COPD** **Curr- smokers**	[11/7]	56(30–72)*	39(8–80)*	71(41–102)*	62(42–69)*	54(24–81)**	36(16–54)**	0.25(0.04–1.65)	90(58–98)*	3 (1–11)**	11 (0–41)
**COPD** **ex-smokers**	[8/4]	65(46–73)*	40(10–179)*	60(47–89)*	58(28–66)*	47(14–84)**	31(10–56)**	0.30(0.03–0.55)	86(10–96)	7 (2–20)	7 (0–20)

Data shown as median (range). Abbreviations*:* COPD: chronic obstructive pulmonary disease, FEV1: forced expiratory volume in 1 second, FVC: forced vital capacity. WCC: total leukocyte count, Volume (mL): the recovered volume of BAL, Volume (% instilled): % volume recovered from instillation of 150 mL, Mac: alveolar macrophages, Lymp: lymphocytes, Neut: neutrophils.

* *p*<0.05;

** <0.001 vs. control.

### Reagents and Antibodies

For assessment of CD98 expression, CD98-PE (BD Biosciences, San Jose, CA) and CD14 and CD45 [phycoerythrin cyanide-5 (PC-5)] (Immunotech/Coulter) were employed. For assessment of the efferocytosis function of human AM, CD33 [phycoerythrin cyanide-5 (PC-5)] (Immunotech/Coulter, Marseille, France) and mitotracker red (Molecular Probes, Oregon, USA) were employed. For assessment of efferocytosis in a MH-S cell line, MHC II (I-A/I-E) [fluorescein isothiocyanate [FITC)] (eBiosciences, San Diego, California, USA) and mitotracker red were used. Recombinant human galectin-3 was produced and purified as previously described [23]. For the Rac1 pull-down assay, anti-Rac1 and anti-Cdc42 were obtained from Millipore. For actin studies, rhodamine phalloidin was obtained from Molecular Probes. Leupeptin, aprotinin, NaF, Na_3_VO_4_ and 2-vinylpyridine were obtained from Sigma Chemical Company Ltd (St Louis, Missouri, USA). Β-actin antibody was purchased from Cell Signaling (Danvers, Massachusetts, USA).

### ELISA Measurement of Galectin-3

Galectin-3 levels were determined in batches of frozen BAL samples (after removal of cells) from subjects with COPD and control subjects using commercial ELISA kits (R&D Systems (Minneapolis, MN)) following the procedure recommended by the manufacturer. Galectin-3 concentration was expressed as nanograms of protein/mL. The mean minimum detectable dose was 0.016 ng/mL.

### Flow Cytometric Analysis of CD98 on AM

Flow cytometry was utilized to assess expression of CD98 on AM from a cohort of subjects as previously reported [3].

### Efferocytosis Assay

Flow cytometry was utilized to assess the ability of AM to phagocytose apoptotic bronchial epithelial cells as previously reported [2]–[4].

### Cell Lines

A murine alveolar macrophage cell line (MH-S; CRL-2019; American Type Culture Collection, Manassas, VA) was maintained in RPMI-1640 medium supplemented with 10% FCS, sodium pyruvate (1 mM), L-glutatmine, HEPES (10 mM), sodium bicarbonate (1.5 g/L), β-mercaptoethanol (0.05 mM) and gentamycin. Normal mouse mammary gland epithelial cells (NMuMG) were maintained in DMEM (Gibco) Invitrogen, Auckland, New Zealand) supplemented with 10% FCS, 10 µg/mL insulin, 100 U/mL penicillin, and 50 µg/mL streptomycin. 16 HBE bronchial epithelial cells for the efferocytosis assay were maintained as previously reported [2].

### Effect of Galectin-3 on Efferocytosis in vitro

AM collected from 4 of the control subjects and 7 subjects with COPD were incubated with galectin-3 (50 or 100 µg/mL) or media control for 10 min at 37°C in serum free media before the addition of apoptotic airway epithelial cells (ratio 4∶1) and assessment of efferocytosis as previously reported [2]–[4]. To address if these effects were mediated by its carbohydrate-binding domain we tested the effect of galectin-3 on efferocytosis in the presence of lactose (5 mM), a disaccharide, competitive for carbohydrate-binding by galectin-3.

### Effect of Galectin-3 on Arginase-1 Activity

Activity of arginase-1, a marker of alternative macrophage activation, was measured in supernatants from AM collected from the 5 healthy controls that had been incubated with galectin-3 for 48 h, using a commercial QuantiChrom TM Arginase Assay Kit (BioAssay Systems, Hayward, CA, USA) as previously described [4].

### Intracellular Glutathione-redox Assays

The glutathione-redox is the ratio of reduced glutathione (GSH) to oxidized glutathione (GSSG). GSH and GSSG levels were measured as we have previously described [24]. Briefly, MH-S macrophages were treated varying concentrations of galectin-3 for 2 h and suspended in ice-cold extraction buffer (1% Triton X-100 and 0.6% sulfosalicyclic acid) and lysed by repeated freezing and thawing. Lysates were centrifuged (10,000×g) for 5 min at 4°C before measurement of GSH and GSSG. Oxidized Glutathione (GSSG) was measured after derivatizing the reduced GSH with 2-vinylpyridine (1 M). Oxidized GSH standards were used and the GSSG-to-GSH ratio calculated.

### Involvement of PI3K in Galectin-3 Induced Efferocytosis

MH-S macrophages were seeded in 24-well plates at 2×10^5^ cells/well and incubated overnight. Cells were pre-treated with the PI3K inhibitor wortmannin (5–50 nM) for 30 min at 37°C, prior to treatment with galectin-3. The efferocytosis assay was performed using NMuMG mouse epithelial cells as phagocytic targets as previously reported [16].

### Effect of Galectin-3 on Rac Activation

Rac pull-down activity assays were performed according to the manufacturer’s indications (Millipore). Briefly, 5.0×10^6 ^MH-S macrophages were plated overnight in reduced serum conditions and were exposed to galectin-3 (50 µg/mL) for 10 min prior to the addition of apoptotic NMuMG cells. Non-ingested cells were removed. Cell extracts were prepared at 4°C from equal amounts of cell protein, using prechilled Mg^2^ lysis buffer (125 mM HEPES, 750 mM NaCl, 5% Igepal CA-630, 50 mM MgCl2, 5 mM EDTA and 10% glycerol; with the following inhibitors: 10 µg/mL leupeptin, 10 mg/mL aprotinin, 1 mM sodium orthovanadate, and 1 mM sodium fluoride). Extracts were cleared by centrifugation for 10 min at 4°C in the presence of glutathione agarose. Fifty µL samples were withdrawn for analysis of total Rac1 and β-actin to ensure equal loading. The remainder of each extract was incubated for 1 h at 4°C with 10 µL of p21-activated kinase-PBD agarose (50% slurry), which selectively binds the active or GTP-loaded forms of Rac and Cdc42. The beads were washed three times with assay buffer and eluted with SDS-PAGE sample buffer. The eluates were then subjected to immunoblot analysis using Rac1-specific antibody.

### Effect of Galectin-3 on Actin Polymerization

MH-S cells were seeded in 8-well chamber slides (BD Biosciences, Bedford, MA, USA) overnight and treated with galectin-3 for 10 min prior to the addition of UV-irradiated apoptotic epithelial cells that been labeled with 200 nM MitoTracker Green [2]. Non-ingested cells were removed and cells were fixed with 4% paraformaldehyde. Cells were permeabilized with 0.1% Triton X-100. Cells were stained with rhodamine-phalloidin for 20 min at room temperature in the dark. Coverslips were mounted with Moval and images were captured using a Bio-Rad Radiance 2100 confocal microscope (at Detmold Imaging Core Facility, Hanson Institute) and Biorad LaserSharp 2000 software for image viewing and processing. In some experiments, wortmannin was added to assess the effects of inhibition of PI3K on actin polymerization.

### Statistical Analysis

The Kruskall-Wallis and Mann Whitney U tests were applied to analyze the data. Analyses were performed using GraphPad Prism software; *p*<0.05 was considered significant.

## Results

### BAL Galectin-3 Levels and AM CD98 Expression are Decreased in COPD

**Figure 1:**
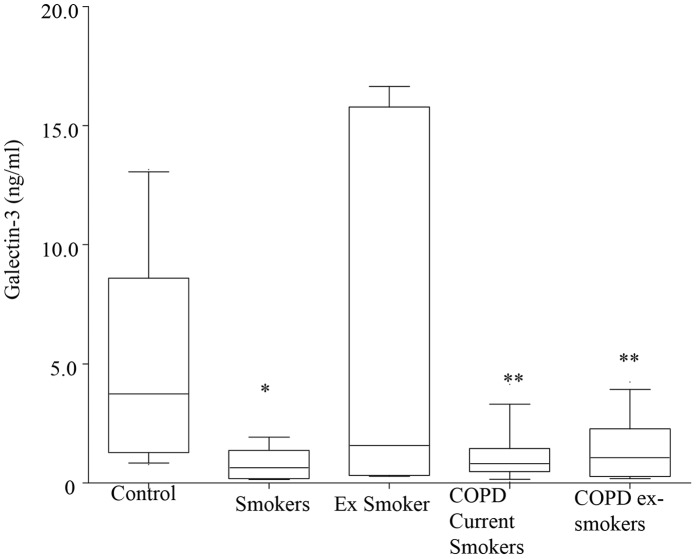
Decreased levels of galectin 3 in BAL in COPD. Galectin-3 analyzed by ELISA in stored samples of BAL from never-smoker controls (n = 15), healthy smoker controls (n = 12), healthy ex-smoker controls (n = 8) and 30 subjects with moderate severity COPD (18 current-smokers and 12 ex-smokers).Box plots present median±25th and 75th percentiles with the 10th and 90th percentiles shown by whiskers outside the box. *p<0.05; **p<0.01 vs. never smoker controls.

**Figure 2:**
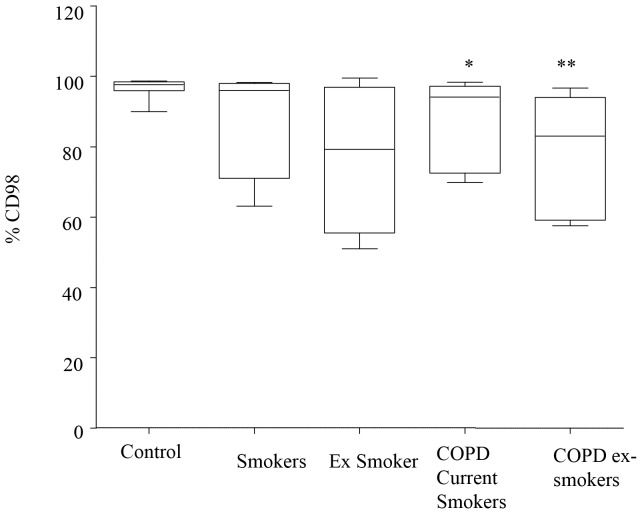
Decreased **AM expression of CD98 in COPD.** Percentage of alveolar macrophages (AM) expressing CD98. CD98 was measured by flow cytometry on AM from 8 never-smoker control subjects and 11 subjects with COPD (6 current smokers and 5 ex-smokers). Data presented as box plots as described in Figure 1. *p<0.05; **p<0.01 vs. never smoker controls.

Galectin-3 levels were significantly decreased in the BAL of both current and ex-smoker COPD and healthy smoker subjects, compared to controls; but not in healthy ex-smokers (Figure 1). There was a small but significant decrease in percentage of AM expressing CD98 from both COPD groups vs. controls (Figure 2) and in mean fluorescence intensity of staining (data not shown). There was no significant correlation between galectin-3 or CD98 and age (not shown). There were no significant associations between BAL yield and levels of galectin-3 (correlation 0.073), and no significant differences in galectin-3 levels between the low recovery group (less than 30% instilled) versus higher recovery group (greater than 30%) BAL (p = 0.291).

### Galectin-3 Increases Efferocytosis, in vitro

**Figure 3:**
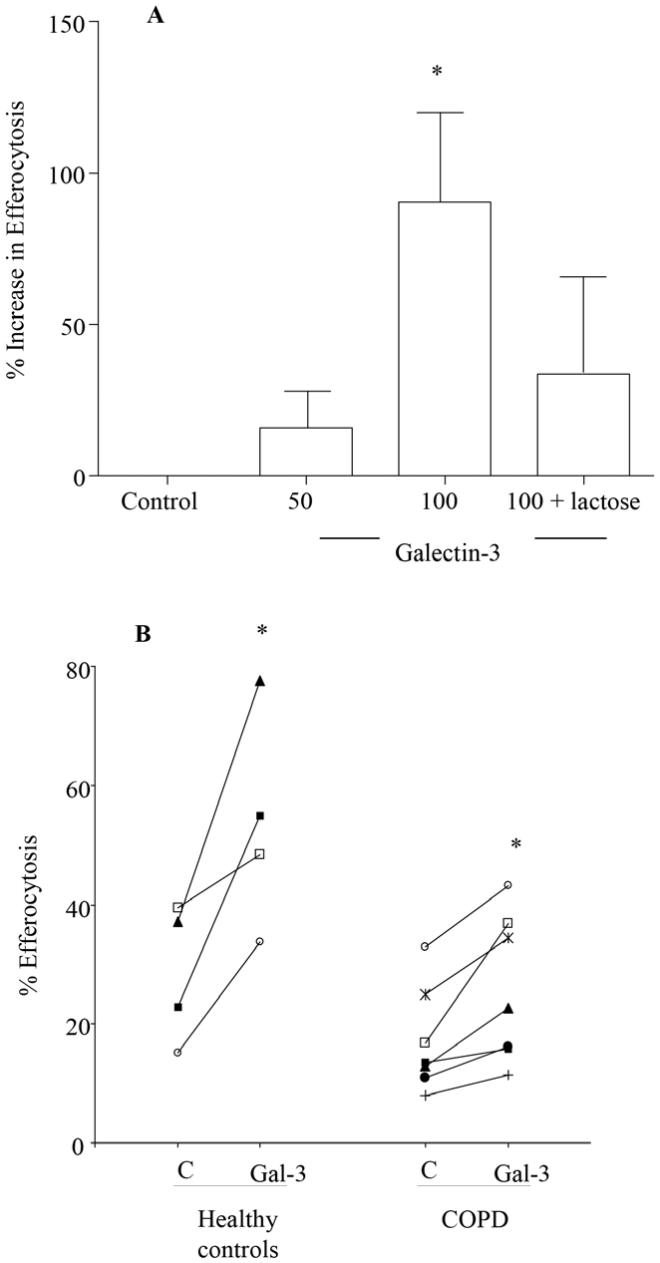
Galectin-3 increases efferocytosis *in vitro*. AM from 4 control subjects and 7 subjects with COPD were incubated with or without 100 µg/mL galectin-3 (Gal-3) for 10 min prior to efferocytosis assay. To address if these effects were mediated by its carbohydrate-binding domain we also tested the effect of galectin-3 on efferocytosis in the presence of lactose (5 mM). (A) Data presented by box plots as described in Figure 1. *p*<0.01 *versus* control with no galectin-3 added. (B) Individual data points showing % efferocytosis pre- and post- treatment with 100 µg/mL Gal-3.

Addition of recombinant human galectin-3 to AM *in vitro* resulted in a dose-dependent increase in efferocytosis (77% increase with 100 µg/mL, *p*  = 0.0028) which was inhibited in the presence of lactose (Figure 3), showing that the stimulatory effects of galectin-3 were mediated by its carbohydrate-binding domain.

### Galectin-3 Increases AM Arginase-1 secretion

**Figure 4:**
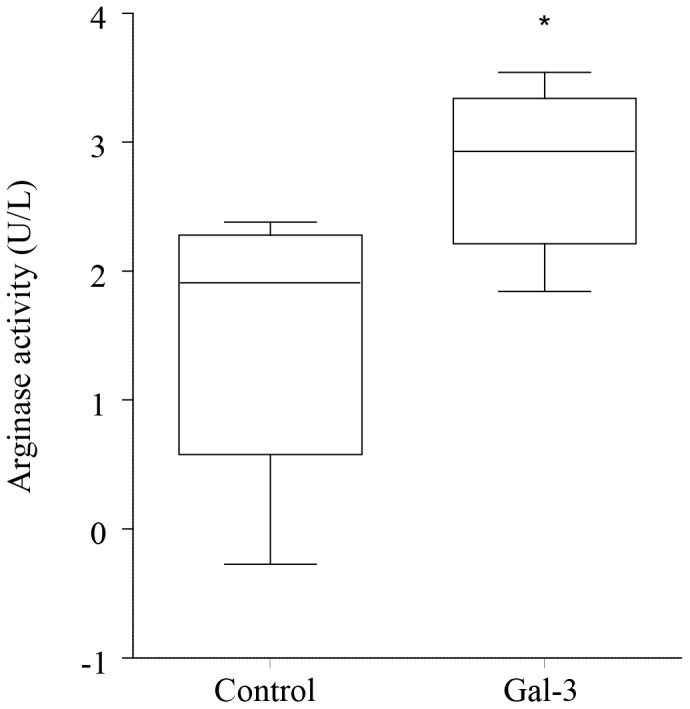
Galectin-3 increases AM arginase secretion. AM from 5 healthy controls were treated with galectin-3 (100 µg/mL) for 48 h. Arginase was measured in culture supernatant by ELISA. *p*<0.05 *versus* control with no galectin-3 added.

There was a significant increase in levels of the alternative activation marker (‘M2’), arginase-1, following treatment of AM with 100 µg/mL galectin-3 for 48 h (Figure 4).

### Galectin-3 Improves Available GSH

**Figure 5:**
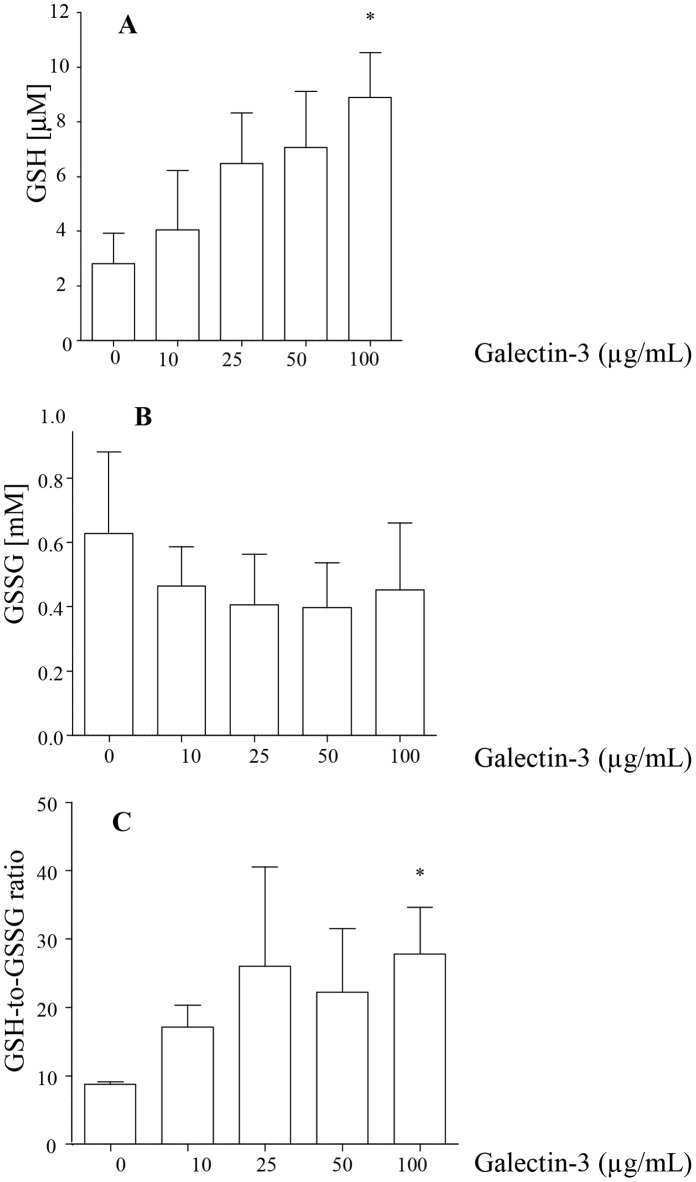
Galectin-3 increases available glutathione. MH-S macrophages were treated with varying concentrations of galectin-3 for 1 h. Cells were lysed and intracellular concentrations of GSH (A) reduced (available) GSH (B) oxidized GSH (GSSG) and (C) the ratio of GSH to GSSG were measured following enzymatic recycling assay. Data represent mean ± SEM of 4 separate triplicate experiments. *p<0.05 *versus* control.

Treatment of MH-S cells with galectin-3 increased GSH and the GSH/GSSG ratio in a dose-dependent manner, reaching statistical significance at 100 µg/mL (Figure 5).

### Galectin-3 Improvement in Efferocytosis is Mediated by PI3K

**Figure 6:**
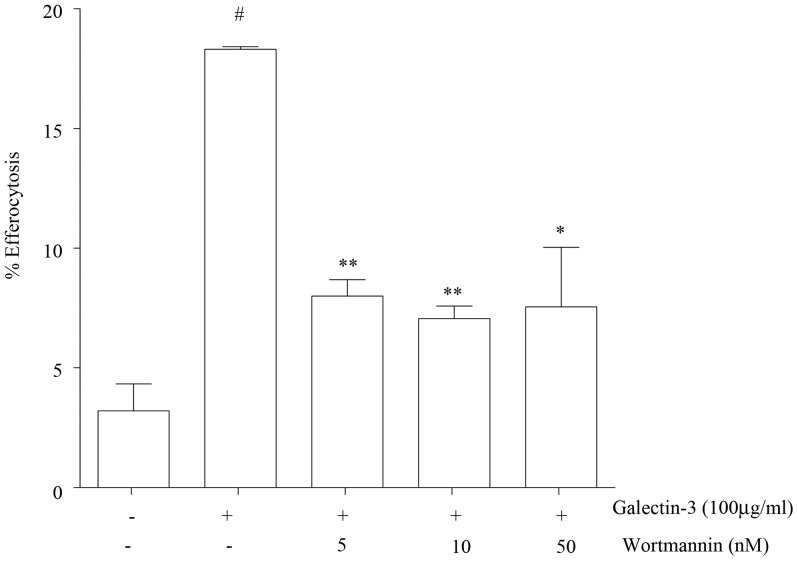
Galectin-3 improves efferocytosis by effects on PI3K. MH-S cells were treated with different concentrations of the PI3K inhibitor (wortmannin) prior to the addition of galectin-3 and assessment of efferocytosis. Data represent mean ± SEM of 3 separate triplicate experiments. *p<0.05; **p<0.01, versus galectin-3 with no wortmannin added.

MH-S macrophages were pre-treated with wortmannin (5–50 nM) for 30 min prior to addition of 100 µg/mL galectin-3 and apoptotic targets. Wortmannin significantly decreased the effects of galectin-3 on efferocytosis (Figure 6).

### Galectin-3 Improvement in Efferocytosis is Mediated by PI3K and Rac1 Activation

**Figure 7:**
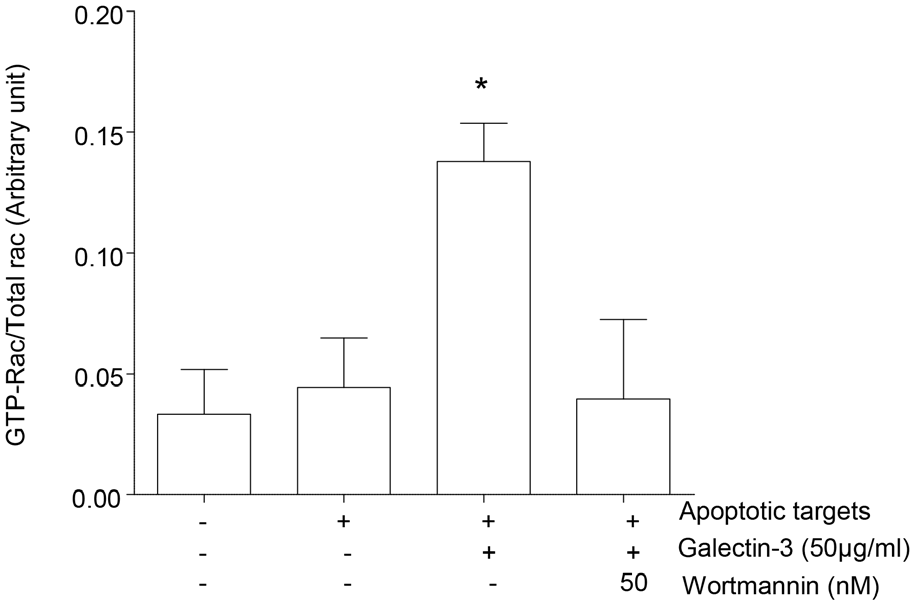
Involvement of PI3K in galectin-3 induced Rac1 activation. Serum-starved MH-S cells were pre-treated with 50 nM wortmannin or diluent for 30 min before the addition of galectin-3 (50 µg/mL) and apoptotic target cells. Active GTP-bound form of Rac1 protein was isolated by pull-down assay and compared to total Rac1 expression was used as a loading control. Data represent 4 separate triplicate experiments. The lanes of the blots (top) correspond with the legends given below the bar diagram. *p<0.05 versus galectin-3 with no wortmannin added.

We assessed Rac1 activation in MH-S cells pre-treated with or without the PI3K inhibitor wortmannin, and then challenged with apoptotic cells in the presence of galectin-3. The active GTP bound form of Rac1 was detectable in cells incubated with apoptotic targets and significantly increased in the presence of galectin-3 (Figure 7). Pre-treatment of cells with wortmannin completely abolished the formation of active GTP-bound form of Rac1. Note that the total amount of Rac1 protein was similar in the treatment groups; this was further confirmed with ß-actin probing indicating that GDP-GTP exchange was inhibited by wortmannin (Figure 7).

### Actin Rearrangement Induced by Galectin-3 is Inhibited by Wortmannin

**Figure 8:**
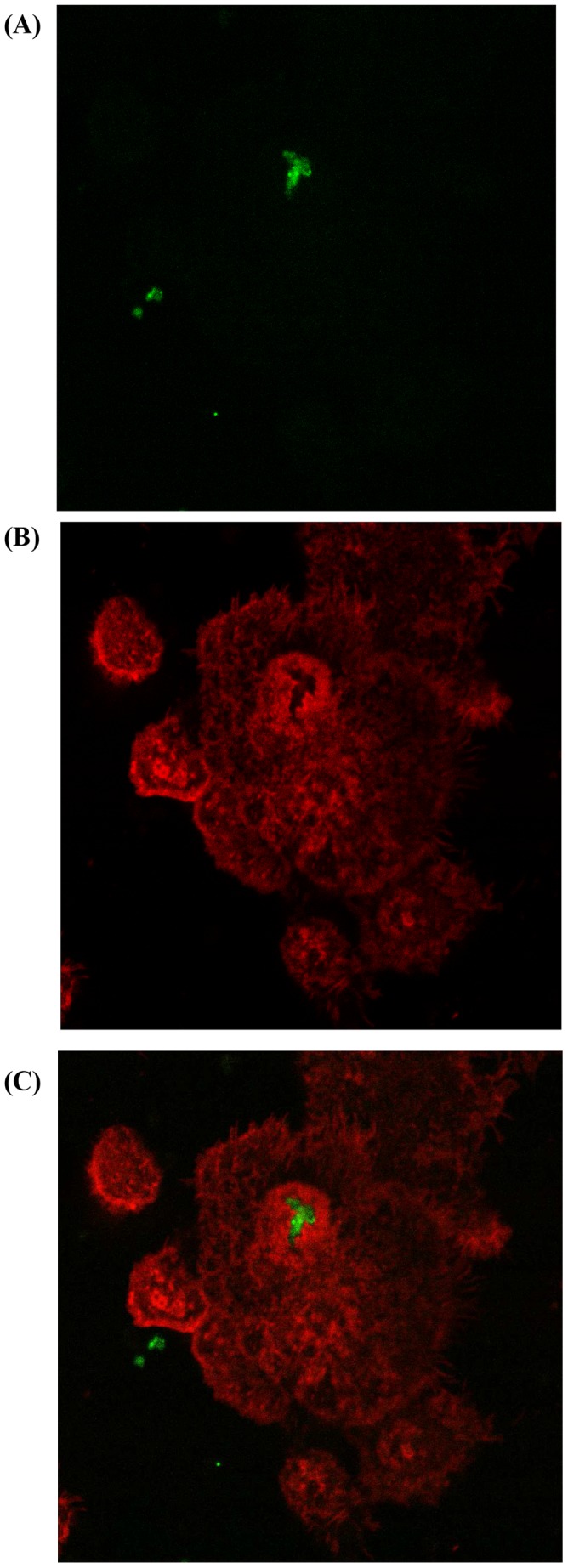
Co-localization of apoptotic cells in F-actin rich phagocytic cups and phagosomes in galectin-3 treated macrophages. MH-S macrophages were pre-treated with 50 µg/ml galectin-3 for 10 min before the addition of MitoTracker Green (MTG) labelled apoptotic epithelial cells. After the indicated time periods, un-ingested apoptotic targets were removed and the cells were fixed, permeabilized, and treated with rhodamine-phalloidin to stain actin. (A) Distribution of ingested apoptotic epithelial cells and (B) F-actin inside macrophages. (C) An overlay of these images demonstrates F-actin surrounding the apoptotic targets.

**Figure 9:**
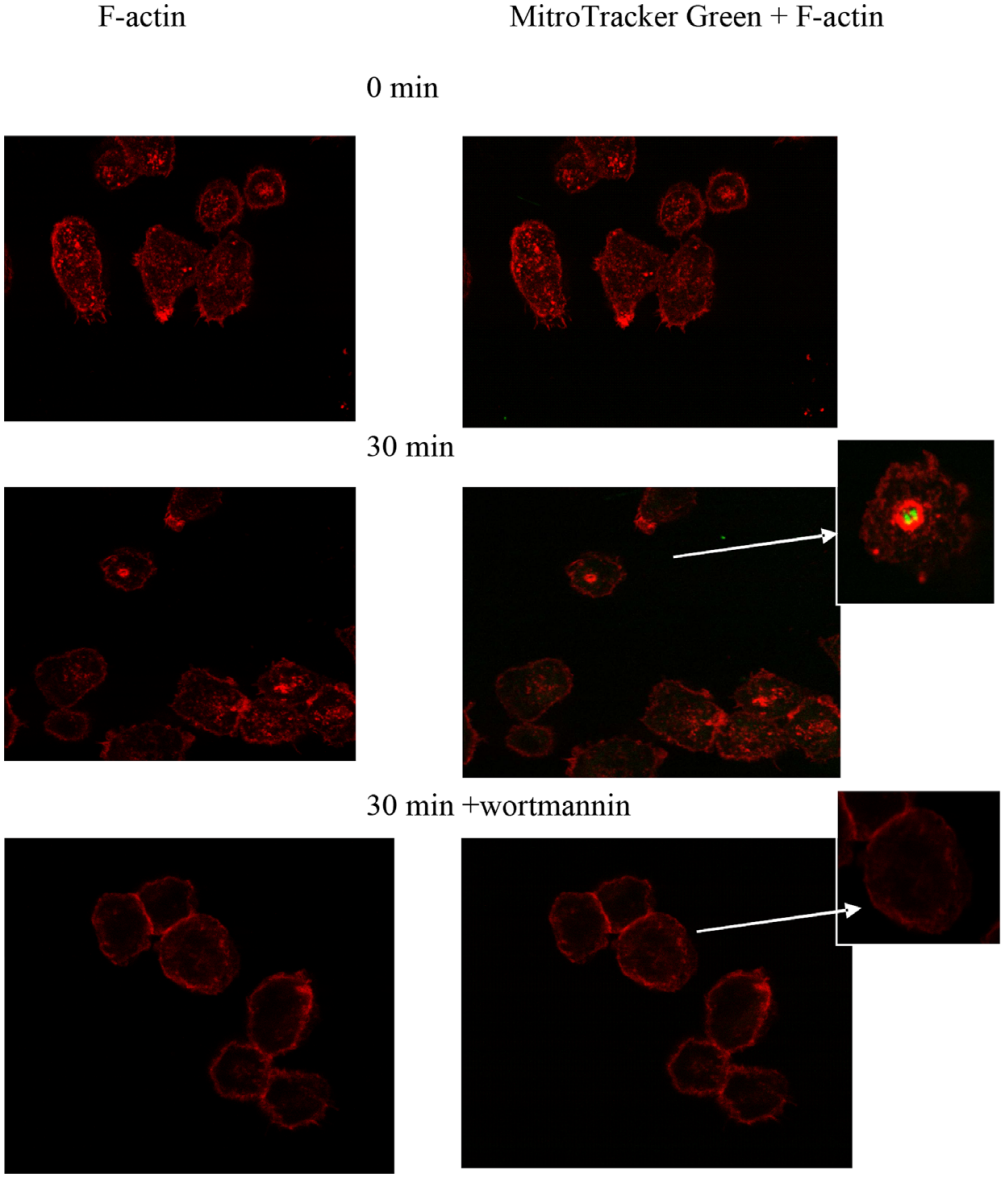
Role for PI3K in F-actin rich phagocytic cups and phagosomes in galectin-3 treated macrophages. MH-S cells were pre-treated with wortmannin before challenge with labeled apoptotic cells in the presence or absence of galectin-3. The cells were then fixed and stained with rhodamine-labeled phalloidin. The appearance of the actin-rich areas was noticeable after 30 min incubation. PI3K inhibition by wortmannin attenuated phagocytosis and the formation of actin-rich phagocytic cups.

MH-S cells were pre-treated with wortmannin before challenge with labeled apoptotic cells in the presence or absence of galectin-3. The cells were then fixed and stained with rhodamine-labeled phalloidin. Co-incubation of MH-S cells with galectin-3 and apoptotic cells induced the formation of actin-rich phagocytic cups around the ingested apoptotic targets (Figure 8). The appearance of the actin-rich areas was noticeable after 30 min incubation (Figure 9). PI3K inhibition by wortmannin attenuated phagocytosis and the formation of actin-rich phagocytic cups (Figure 9).

## Discussion

Our data indicates a potential role for extracellular galectin-3 in the failed efferocytosis that occurs in COPD, and shows that the mechanisms include effects on cytoskeletal remodelling, macrophage phenotype and GSH availability. Both galectin-3 and its receptor CD98 were decreased in the airways of both current and ex-smoker COPD. Galectin-3 was also significantly reduced in BAL from healthy smokers, indicating at least a partial potential effect of cigarette smoke. However, consistent with our findings with MBL, we also noted significant changes in ex-smoker COPD subjects who had ceased smoking indicating that the defects continue once COPD is established, despite cessation of smoking. A significantly lower volume of BALF was recovered from both current and ex-smokers with COPD, compared to healthy controls, consistent with our previous studies. It should be noted that low recovered BAL fluid tends to contain less alveolar lining fluid, potentially resulting in relatively low concentrations of soluble mediators and more neutrophils (that could express/interact with CD98). We did not, however, note any differences in the percentage of neutrophils in BALF from any group.

Previous studies from our group and others have highlighted the importance of deficiencies in BAL levels of soluble lectins including MBL in the pathogenesis of COPD [14]. Only one study implicated a role for galectin-3; intracellular expression was increased in the small airway epithelium and alveolar macrophages of severe COPD patients [25]. While this appears contradictory to our results, the pleiotrophic functions of galectin-3 are important in the interpretation of these results. There is a significant amount of evidence which demonstrates that biological effects of galectin-3 depend on its intracellular or extracellular location. Extracellular galectin-3 has been shown to induce apoptosis in activated T-cells [26], while intracellular galectin-3 protects cells from apoptosis and promotes their proliferation [27].

Interestingly, both MBL and galectin-3 bind to CD98 [8], [21], and the interaction of galectin-3 and CD98 has been shown to induce an ‘M2’ alternatively activated macrophage phenotype [21] (with improved ability to phagocytose apoptotic cells). The reduced expression of galectin-3, MBL and CD98 are thus consistent with our previous findings of a mixed but predominantly ‘M1’ phenotype in the airways in COPD, with reduced efferocytic ability. We showed that the pro-efferocytosis effects of galectin-3 are likely to involve its effects on macrophage phenotype, as galectin-3 was able to steer AM to a more ‘M2’ phenotype, evident by an increase in arginase release.

Previous studies have demonstrated a role for lectins, including galectin 3, in host defence against pathogens by promoting opsonisation by complement and/or pathogen clearance by host macrophages [28]–[30]. The uptake of beads in the presence of galectin-3 could be prevented by the addition of lactose, via mechanisms that include carbohydrate recognition [30]. We have now shown that exogenous galectin-3 also improved efferocytosis via similar mechanisms. While the dose of galectin-3 used in our studies was quite high, previous studies have shown that levels of extracellular galectin-3 can be as high as 50 µg/mL in BAL after infection in murine models [31], and we did not find any evidence of cell death as a result of galectin-3 treatment in our study.

In this study we were particularly interested in dissecting the mechanisms for the effects of lectins on efferocytosis. Rac1 is a small GTPase which regulates the reorganization of the actin cytoskeleton by activating various effector molecules [32]. We previously showed that the pro-efferocytosis effects of MBL were accompanied by an increase in intracellular Rac1/2/3, suggesting an effect on the actin reorganization that occurs during the phagocytic process [16]. In the present study we showed that the efferocytosis-enhancing effects of galectin-3 were accompanied by reorganization of actin and an increase in active Rac. These changes were significantly attenuated by the inhibition of PI3K, not surprising given the pivotal role that PI3K plays in remodeling of the actin cytoskeleton, enabling the formation of phagocytic cups and, subsequently, the ability of the phagosome to internalize the apoptotic cell target [21]. It should be noted that the attenuation was only partial, implicating the potential involvement of further factors in the galectin-3 effects. Interestingly, a recent study investigating phagocytosis of degenerated myelin by microglia also showed that galectin-3 improved phagocytosis by upregulating and prolonging K-Ras-GTP dependent PI3K activity and also by upregulating expression of complement 3 and scavenger receptor AI/II [17]. Extending our studies to include other inhibitors and assessing the effects of galectin-3 on arginase activity using AM from COPD subjects would provide additional interesting data.

We then showed that a second potential mechanism may be the effect of lectins on intracellular production of glutathione (GSH). We have showed decreased GSH availability in COPD airways [4] and confirmed with our smoking murine model the role of GSH in the failed efferocytosis in COPD [4]. We now demonstrate that galectin-3 improves available intracellular macrophage GSH. Galectin-3, as an endogenous ligand for CD98 [8], [21], when present at normal (high) concentrations could cross-link xCT/CD98 and increase intracellular cystine and its conversion to cysteine and thus increase synthesis of GSH [20,33]. The low levels of galectin-3 and reduced expression of CD98 in COPD subjects are thus likely to compromise the levels of GSH and subsequent efferocytosis ability of airway macrophages, and play a role in the defective efferocytosis. In summary, our data implicate a role for galectin-3 in the macrophage dysfunction that occurs in COPD. Further studies will be important for a better understanding of the links between galectin-3, smoking and COPD.
